# The economic costs and health-related quality of life of people with HIV/AIDS in the Canary Islands, Spain

**DOI:** 10.1186/1472-6963-9-55

**Published:** 2009-03-30

**Authors:** Julio Lopez-Bastida, Juan Oliva-Moreno, Lilisbeth Perestelo-Perez, Pedro Serrano-Aguilar

**Affiliations:** 1 Evaluation and Planning Unit, Canary Island Health Service, Spain; 2 University Hospital Nuestra Sra de la Candelaria, Canary Island Health Service, Spain; 3 University Castilla La Mancha, Madrid, Spain; 4 CIBER de Epidemiología y Salud Pública (CIBERESP), Spain

## Abstract

**Background:**

The objective was to determine the economic burden, as well as the impact on HRQOL for people with HIV/AIDS in Spain in 2003.

**Methods:**

A cross-sectional study of 572 people with HIV were recruited from outpatient clinics in the Canary Islands, Spain. Demographic, health resources utilization, indirect costs and quality of life data were collected through medical records and questionnaires filled out by people with HIV. HRQOL was measured with two generic questionnaires: SF-36 and EQ-5D.

**Results:**

In 2003 annual costs of caring for patients with asymptomatic HIV, symptomatic HIV and AIDS were €10,351, €14,489 and €15,750, respectively. The HRQOL with the EQ-5D was 0.78. SF-36 summary results for physical and mental health were 48.30 and 38.80, respectively.

**Conclusion:**

HIV/AIDS represent a high economic impact from society point of view. the structure of health care costs have changed due to these new drugs, increasing the weight of pharmaceutical treatment over total costs and decreasing the importance of inpatient care costs. In spite of the therapeutic improvements, labour losses/indirect costs still represent a high cost. Costs and HRQOL were strongly associated with severity. Although the latest drug developments have not yet been able to find the definitive cure, they have allowed an improvement in expectancy of life and in the HRQOL of the patients.

## Background

Since the discovery of human immunodeficiency virus (HIV) at the beginning of the 1980s, HIV/AIDS has been one of the greatest health problems in the world [1]. HIV/AIDS places an increasing burden on the health of the population, and causes further socio-economic problems for individuals, families, communities and governments in many countries [2,3].

Since the beginning of the epidemic, 67,466 cases of AIDS have been registered in Spain, with a number of estimated deaths ranging between 40,000 and 50,000. Spain has the highest rate of HIV among Western European countries. The estimated number of people infected by HIV is about 130,000 (range 110,000–150,000). However, it is thought that only 75% of all cases have been diagnosed and receive appropriate treatment. Epidemiological studies in Spain show that there are approximately 97,500 people diagnosed with HIV/AIDS [4]. National information on the HIV/AIDS disease severity distribution (39.5% asymptomatic HIV, 19.4% symptomatic HIV and 41.1% AIDS) enables us to distribute these people into the following three groups: 38,513 as asymptomatic HIV, 18,915 as symptomatic HIV and 40,073 as AIDS [5].

In high-income countries, the medical advances of recent years have yet to produce a cure, although they have been able to increase the life expectancy and improve the quality of life of the virus carriers, as well as delay the terminal stage of the disease [6]. As a consequence of advances in therapy since the mid-90s, several reports have documented a shift in HIV/AIDS health service utilization from inpatient to outpatient care in developed countries since the introduction of Highly Active Antiretroviral Therapy (HAART) [7,8]. Most economic assessments carried out in this area deal with the determination of direct costs (prevention, diagnosis and treatment of HIV disease). Economic assessments incorporating the costs associated with productivity losses (indirect costs) are exceptions in the health economics literature on HIV [9,10]. It is important to stress that most of the people with HIV in Spain are of working age (82% being between the ages of 25 and 39 years), and have been forced to leave the labour market because of their illness [5]. However, the effects of antiretroviral treatments on patient survival and reduced morbidity improve the chances of people to participate in the labour market.

Assessing health-related quality of life (HRQOL) is useful for documenting the patient's perceived burden of chronic disease, tracking changes in health over time, assessing the effects of treatment and quantifying the return on health care investment [11]. The reduction in HRQOL relative to the progression of the disease is also relevant for the allocation of health care resources, and should be taken into consideration by the clinicians treating people with HIV.

In spite of Spain's relatively high HIV prevalence rate (when compared to other European countries), there have been no recent studies on HIV-related costs in Spain. The aim of this study was to calculate the economic costs of people with HIV/AIDS in the Canary Islands, Spain. We also examined the HRQOL of people with HIV/AIDS.

## Methods

### Research field and subjects

It was carried out a cross-sectional, retrospective study of people diagnosed with HIV/AIDS receiving outpatient care. Patients were recruited according to disease severity from four hospital outpatient clinics in the Canary Islands, Spain. The 1993 classification scheme proposed by the Centre for Disease Control and Prevention (CDC) in the United States to divide the disease into HIV-asymptomatic, HIV-symptomatic and AIDS stages [12] was used to establish the severity of HIV infection.

All patients were informed about the study objectives and the confidentiality of the data and were asked to show their agreement with, and their understanding of, the study conditions by signing a declaration of consent form. The study was approved by the ethics research committee of University Hospital Nuestra Sra. de la Candelaria.

### Information and variables of interest

The fieldwork was carried out between January and December 2003. Demographic and clinical data were collected for people previously diagnosed with HIV/AIDS. The patients recruited were at least 18 years old and they were treated in outpatient clinics.

Apart from those who did not agree to take part in the study, incarcerated HIV people were not included in the analysis. Also, some patients were excluded from the final analysis when the information gathered was incomplete.

The information sources used in the study were the patients' medical records and self-completed questionnaires. We used two different questionnaires to collect data about the direct and indirect costs and HRQOL. Patients were asked about their employment and wages when HIV infection was diagnosed, as well as their current situation, in order to collect data for the calculation of the indirect costs.

HRQOL information was collected only once using two generic questionnaires: EQ-5D and SF-36.

### Cost Methodology

We used the prevalence approach. The prevalence of the disease takes into account all of the existing cases during a given year and all of the resources used for prevention, treatment and rehabilitation, plus the productivity losses as a result of morbidity within that year. The cost of illness based on prevalence has the advantage of incorporating the measurements of total annual health care expenditure, which is particularly relevant for a chronic disease like AIDS where long-term treatment is needed [13].

A bottom-up costing approach was used, which normally begins from a defined subpopulation with a certain pathology and records every cost of the disease related to the disease pathology [14]. The point of view used here for the analysis is from the societal perspective, and the unit prices relate to 2003.

### Direct health-care costs

Direct costs were derived from health care utilization. The resources used by each patient were given a value, in terms of the relevant unit costs and the average cost per patient in the sample, as well as in terms of the average cost per patient at various levels of severity (asymptomatic, symptomatic, AIDS and CD4 cell count). Unit prices should reflect opportunity cost, although in practice other cost measurements (like accounting cost) are often used.

Information about the number of hospital admissions, medical tests and use of prescription drugs for problems related to HIV/AIDS was taken from medical records. Unit costs came from a number of available sources and were based on a costing exercise. Inpatient care was based on Diagnosis Related Group (DRG) costs. Unit costs (not charges for reimbursement) for DRGs were valued on the basis of the average costs per DRG from 18 hospitals in Spain (one hospital from the Canary Islands was included) [15].

Prescription drug costs were calculated using data from the hospitals. The hospital drug costs were based on the official selling price to hospitals in the Canary Islands.

We used information obtained through questionnaires filled out by the patients with HIV/AIDS to determine the number of emergencies, rehabilitations and medical visits related to HIV/AIDS.

The mean costs per emergency visit, rehabilitation and medical visit were taken from the SOIKOS health care unit cost database [16]. SOIKOS is the most complete database used in Spain to obtain health care unit costs. The sources of the unit costs are published articles, reports, hospital accounting systems, etc., and the figures are updated every year.

### Indirect costs

The survey included questions about labour participation and individual wages, firstly, when the person received the HIV's diagnosis and, secondly, at the time of answering the questionnaire. The theoretical basis used for the estimation of the labour losses was the human capital approach [17]. Based on this approach, productivity losses can be estimated through changes in labour status and in wages.

### Patient outcomes

Two types of patient outcomes were examined in the analysis: 1) health status, as measured by the physical and mental health summary scores from the Medical Outcomes Study Short-Form (SF-36) [18], and 2) health utilities, as measured by the EQ-5D [19]. Both are well known HRQOL generic instruments and are used to measure HRQOL among the general population as well as in a variety of different chronic diseases.

The Short Form-36 Health Survey (SF-36) is one of the most widely used and evaluated generic health-related quality of life (HRQOL) questionnaires and has already been validated and is in widespread use in Spain [20,21]. SF-36 consists of 36 questions [18], which are categorised into 8 subscales that can be subdivided into two categories: physical and mental health measures.

EQ-5D is a simple generic instrument developed by a multidisciplinary group of researchers [19]. EQ-5D has been validated in Spain, and it is commonly used in the area of economic evaluation and technology assessment. In two regions of Spain (Catalonia and the Canary Islands) EQ-5D is used for periodic health surveys where the two populations are similar [22,23]. There are five questions in EQ-5D covering the areas of mobility, self care, everyday activities, pain/discomfort and anxiety/depression. A total of 243 possible health states can be defined in this way. Valuations of these health states are given for the general population [24]. The values, or utilities, are scaled such that 0 is the value of death and 1 is the value of perfect health.

## Results

### Patients' characteristics

A total of 578 questionnaires were collected from people with HIV/AIDS (72.3% of the total sent), 6 of which were excluded because the information contained was insufficient or inadequate. The main characteristics of the sample in terms of age, gender, stages of HIV infection and transmission categories are shown in Table 1 [see Additional file 1]. We have no information on the characteristics of HIV people who did not participate in the study. However, the proportion of patients contacted who did not agree to participate in the study was very low.

The study randomly selected 400 patients from the 572 person who participated in the study to calculate indirect costs. We obtained 241 patients answers (60.3% of the total sent), 4 of which were excluded because the information was insufficient or inadequate. This sub-sample accounted for near of 10% of the estimated HIV cases in the Canary Islands. No significant differences were found between the mean age, sex and clinical variables of this sub-sample and the sample of 572 people with HIV used for the calculations of direct costs [see Additional file 2].

### Results of the costs for people with HIV/AIDS

The mean costs were €10,531, €14,489 and €15,750 for patients with asymptomatic HIV, symptomatic HIV and AIDS, respectively, in 2003 [see Additional file 3]. The health care costs increased with the severity of disease, as expected. Upon analysing the internal distribution of the costs for each of these groups, the drugs were seen to be the largest contributor to health care costs for asymptomatic HIV, symptomatic HIV and AIDS patients. Medical tests were the second highest in terms of importance for asymptomatic HIV and symptomatic HIV patients, whereas hospitalisations were the second highest for AIDS patients. In addition, we calculated health care costs for patients with CD4 cell count [see Additional file 4], for comparison with other studies [25]. The CD4 cell counts were obtained from the latest available data collected from medical records.

### Quality of life of people with HIV/AIDS

Women had lower SF-36 scores than men, with statistically significant differences in social function and emotional role. Patients between 31 and 40 years of age had higher scores in the physical component summary and in four of the eight domains (physical function, social function, vitality and mental health). All of the SF-36 domain scores (except bodily pain) gave different results for asymptomatic HIV, symptomatic HIV and AIDS patients.

The mean physical component summary (PCS) scores were 50.07, 47.44 and 46.07 for people with asymptomatic HIV, symptomatic HIV and AIDS, respectively, in the SF-36. The mean mental component summary (MCS) scores were 39.52, 40.99 and 35.63 for people with asymptomatic HIV, symptomatic HIV and AIDS, respectively.

Symptomatic HIV patients had significantly lower scores than asymptomatic HIV patients in the physical role and general health. Patients with AIDS had lower mean scores in all HRQOL domains than asymptomatic HIV patients and also had lower mean scores in the social function, mental health and mental component summaries than symptomatic HIV patients. Univariate analyses indicated that CD4 lymphocyte counts were significantly correlated with physical functioning (SF-36). A weak and negative correlation was observed between the viral load, general health and physical functioning scores (SF-36).

QOL ratings on both the physical functioning and the psychological well-being aspects of the SF-36 were lower in people with AIDS, and significant differences were obtained for patients according to the severity of HIV disease. It is possible to discriminate, using the dimensions of the SF-36, between patients with HIV and AIDS, especially between patients with asymptomatic HIV and AIDS.

The mean social tariff scores in the EQ-5D were 0.79, 0.80 and 0.73 for patients with asymptomatic HIV, symptomatic HIV and AIDS, respectively. The values of HRQOL differed significantly according to disease severity (P < .001) and showed that the HRQOL scores, as measured by the SF-36 and the EQ-5D, were related to disease severity [see Additional file 5]. Direct and indirect costs, after controlling for HRQOL using EQ-5D, are shown in Table 6 [see Additional file 6].

## Discussion

The introduction of triple drug therapy has represented a new era in the therapeutic management of HIV/AIDS from an economic point of view. It seems clear that the diffusion of new drug therapies has increased the lifetime cost of care in comparison with the early 1990s [25-27], primarily due to an increase in life expectancy of HIV+ people. However, it is not clear whether the HAART therapy has increased the annual health care cost per patient. In Canada Krentz [28] reported an increase of 71% for health care costs between 1995 and 2001 (from 655 to 1119 Canadian dollars monthly), whereas Stoll [29] described a 32% reduction in direct costs between 1997 and 2001 (from €35,865 to €24,482 per year). More recently, Merito [8] also reported direct health care cost reductions for HIV and AIDS management.

These discrepancies could be partially explained by international differences in the various components of the costs. However, in some countries like France the same discrepancy has been reported: Flori & le Vaillant [30] found a decline of 25% in patients' health care costs between 1995 and 2000; meanwhile, Basuyau [31] reported a large increase in these costs when comparing the 1992–1996 and 1996–2000 periods.

An increase in overall costs has been observed in Spain from 1995 [32] to 1997 [33], followed by a reduction from 1997 [33] to our data in 2003. Although costs data for people with asymptomatic and symptomatic HIV in Spain have been very similar in recent studies, the costs for people with AIDS were higher in a previous study [33] compared to our estimations. Cost differences between 1997 and 2003 are due to the reduction in the overall costs for patients in the AIDS stage and, in particular, in costs associated with hospital use.

We use human capital approach for estimating labour productivity losses. Although this is the most commonly method used for measuring and evaluating productivity losses [13], there are alternative methods as the frictional costs approach [34]. In the frictional costs model productivity losses due to premature death, permanent and temporary sick leave are lower than in the human capital approach. In fact, production losses would be zero, from a long-term perspective, after an adjustment or "frictional period".

As a consequence of these different methodological approaches, the empirical results obtained comparing these two methods are very different, with lower values using the frictional costs method [34]. So, it is not surprising that the use of different costing methods can lead to different policy conclusions [35]. We focus on the human capital calculation because this approach has a solid economic tradition and takes a social perspective [36] while frictional costs approach is not exempt from criticism. As Liljas [37] pointed out "...rests on very strong assumptions of the labour market which do not have a foundation in economic theory".

Studies assessing indirect costs are fairly scarce in the international field. Before the therapeutic advances that involved the HAART, the impact of HIV on the labour participation of HIV-infected people was devastating. Scitovsky and Rice [38] assumed that a typical AIDS patient is too ill to work for 60% of his or her time. Yelin [39] reported that half of those working when they were informed of the diagnosis left work within the following two years. Massagli [40] stated that 76% of these people were working at the moment of diagnosis and only 53% at the time of the survey (with an average of 16 months elapsing since the diagnosis). The loss of wages was calculated as being 75% for the whole sample. Leigh [41] concluded that, when compared with a control group (not HIV-infected), patients with an AIDS diagnosis experience a substantial loss of working time, but this is also the case with HIV-infected patients who do not develop the disease. Laursen and Larsen [42] found that 50% of the patients were employed when they were diagnosed with AIDS, 15% were off work and 19% were receiving sickness benefits. The percentage of people in employment fell to 22% a month after diagnosis, and to 6% two years afterwards, with 67% having died. Therefore, the development of the illness reduces the life expectancy of the patients and forces them, in many cases, to leave the job market.

However, therapeutic advances made since the mid 1990s have substantially changed this scenario. Dray-Spira [43], stated that between 46.8% and 58.8% of HIV+ people (depending on the region of France considered) were employed, and a third of those unemployed said they were looking for work in 2001. Rabkin [44] concluded that patients who already had work at the beginning of the follow-up had a higher probability of keeping it. On the other hand, people who were unemployed did not manage to return to their jobs or find another job. Goldman and Bao [45] studied the effect on patients of the use of HAARTs in terms of the probability of returning to work, keeping the same position and number of hours worked. Their results indicate that the probability of keeping a job in the 6 months following the beginning of the treatment goes up from 58% to 94% as a result of the treatment. In addition, they clearly stated that the results are more favourable when the treatment is started in the early phases of the infection. A recent paper by Bernell and Shinogle [46] came to a similar conclusion: HAARTs substantially increase the HIV-infected person's chances of participating in the workforce. Nevertheless, this does not mean that an HIV diagnosis does not affect the patient's participation at work or that its impact is less. However, Auld [47] suggested that the decrease in the percentage of HIV-positive people in employment is a consequence of the adaptation of expectations of the person to a health shock that implies a lower life expectancy. Along the same lines, Auld [47] calculated that the impact of the diagnosis decreases the chances of finding employment by 25%.

In Spain, we are aware of only one pilot study on patients with HIV/AIDS where Oliva et al., [10] found that the better the HRQOL, the higher the probability of being employed. However, in our study, productivity losses were high among patients despite their good HRQOL.

As regards HRQOL, previous studies with comparable data also showed equivalent values for SF-36 physical functioning in people with asymptomatic HIV and the general population, which then worsened with the progression of the disease [11,40]. Comparing this information with our more recent data, we observed better physical functioning SF-36 scores, which could be interpreted as the result of the impact of changes in the treatments (use of new and more effective treatments, different access to medications among countries, etc).

In line with some other studies [11], emotional well-being, measured by SF-36, was significantly worse in people with HIV/AIDS than in the general population in Spain and in people with other chronic diseases (mean (± SD) 39 (± 14) vs. 50 (± 10)). Despite improvement in treatment outcomes, people with AIDS have worse emotional well-being than patients with asymptomatic and symptomatic HIV disease. Co-morbid psychiatric illnesses, including anxiety and depression, were frequent in HIV-positive patients [46]. Previous studies showed comparable levels of emotional well-being among patients in different stages of HIV disease, although the values were significantly worse than for the general population and patients with other chronic diseases [11].

Furthermore, several studies showed a negative relationship between symptoms and health-related quality of life in patients with HIV disease [11,48,49]. These studies support the conceptual model that links clinical measures with health-related quality of life, suggesting that effective clinical management of HIV-related symptoms may improve functioning and well-being in these patients.

In line with our results for SF-36, EQ-5D values in people with HIV/AIDS showed lower age-adjusted scores than for the general population in Spain, but higher scores than for people with certain other chronic diseases, such as neurodegenerative disease, cancer, stroke, heart attack, chronic heart failure and diabetes mellitus. Although generic measures of HRQOL such as SF-36 or EQ-5D have previously been used on people with HIV/AIDS, the interpretation of our data suggests that these generic instruments might lack the sensitivity needed to capture potential HRQOL differences between patients with asymptomatic and symptomatic HIV.

Certain studies compared and commented on the use of the SF-36 and MOS-HIV instruments in studies of persons with HIV disease [50]. Although MOS-HIV is widely used in monitoring HIV-infected persons, it has noteworthy limitations that may constrain its applications to this population [50]. Therefore, there is insufficient evidence in the literature to support the use of MOS-HIV, as opposed to using SF-36, for HIV-infected persons.

## Conclusion

In conclusion, HIV/AIDS represent a high economic impact from society point of view. Our results confirm that the main component of the health care costs in Spain was pharmaceuticals, with a lesser emphasis on inpatient care costs. We also show the strongly and positive association between costs and severity of disease. Annual costs associated with asymptomatic phase were estimated in €10,531, €14,489 associated with symptomatic phase and €15,750 associated with AIDS. In spite of the therapeutic improvements, labour losses (indirect costs) still represent an important economic impact range from a 32.1% (asymptomatic) to 41.3% (symptomatic) of total costs. Although the latest drug developments have not yet been able to find the definitive cure, they have allowed an improvement in expectancy of life of people with HIV and to get a better health related quality of life.

## Limitations of the study

Our results were obtained from a cross-sectional study performed in the Canary Islands (Spain), which may limit generalisation. However, outpatient clinics at the four teaching hospitals in the Canary Islands have been selected to reflect potential differences in diagnostic and therapeutic patterns. The large sample size used in our study for the estimation of health care costs (23% of estimated people HIV-infected in the Canary Islands) and labour productivity losses (10%) ensured that all degrees of disease severity were represented in sufficient numbers to make it possible to obtain consistent results.

Although we have no control group for the estimation of indirect costs, the evolution of the Spanish labour market has been favourable to job creation since the period 1996 until the time of the survey. The effect of the business cycle on the labour market has been clearly positive the last years and can not be considered in general terms that a worsening of labour participation and wages was due to macroeconomic effects. Therefore, the effect found on labour participation and wages can be interpreted as a minimum threshold of labour losses associated to HIV.

The Canary Islands with a population close to 2 million people have a very similar demographic structure, aging rate, health expectations and infant mortality to the average values in Spain [51]. The National Health System in any of the 17 Spanish regions guarantees free access and universal coverage, as well as an identical health care package for every citizen [52]. Furthermore, ratios of health care resources (professionals, structural and technological equipment) in the Canary Islands are in line with the average values in Spain [53]. In addition, the organisation of health care services for HIV infected patients is fairly homogeneous in Spain, with most of the services provided by outpatient clinics or day care clinics [54]. Therefore, there is no reason to assume that the cost of the resources in our study is substantially different from elsewhere in Spain. Further studies are needed to study costs for longer time periods.

## Abbreviations

**HIV**: Human Immunodeficiency Virus; **AIDS**: Acquired Immune Deficiency Syndrome; **HAART**: Highly Active Antiretroviral Therapy; **HRQOL**: Health-Related Quality Of Life; **CDC**: Center for Disease Control and Prevention; **DRG**: Diagnosis Related Group; **EQ-5D**: EuroQol-5D; **SF-36**: Short Form-36 Health Survey; **MCS**: Mental Component Summary; **PCS**: Physical Component Summary.

## Competing interests

This study was supported by an unrestricted educational grant awarded jointly to the Universities of Carlos III de Madrid and Pompeu Fabra de Barcelona by The Merck Foundation, the philanthropic arm of Merck Co. Inc., White House Station, New Jersey, USA; The Canarias Foundation of Health and Research (FUNCIS). File N°: 48/02, and from project SEJ2005-08793-C04-01-04.

## Authors' contributions

JLB conceived of the study and participated in: its design, the data collection, the data analysis, and the draft of the manuscript. JOM, LPP, and PSA participated in the data collection, the data analysis, and to the manuscript revision. All authors have read and approved the final manuscript.

## Pre-publication history

The pre-publication history for this paper can be accessed here:



## Supplementary Material

Additional file 1 **Table 1.** Main characteristics of the sample in the Canary Islands. Click here for file

Additional file 2 **Table 2.** Main characteristics of the sample for direct and indirect costs. Click here for file

Additional file 3 **Table 3.** Mean Cost for people with HIV and AIDS in the Canary Islands, 2003. Click here for file

Additional file 4 **Table 4.** Direct Costs for people with HIV and AIDS in the Canary Islands (2003). Click here for file

Additional file 5 **Table 5.** Mean scores for SF-36 and EQ-5D. Click here for file

Additional file 6 **Table 6.** Direct and indirect costs and quality of life. Click here for file
