# Supplementary material for: The economic costs and health-related quality of life of people with HIV/AIDS in the Canary Islands, Spain
Source: BMC Health Serv Res. 2009 Mar 30;9:55. doi: 10.1186/1472-6963-9-55 (PMC2670289; doi:10.1186/1472-6963-9-55)
Supplement: Additional file 1 — Table 1. Main characteristics of the sample in the Canary Islands. [file 1472-6963-9-55-S1.doc]

**Table 1. Main characteristics of the sample in the Canary Islands**

|  | **Canary Islands (2003)** | | | |
| --- | --- | --- | --- | --- |
|  | **N** | **(%)** | Mean  **(I.C. 95%)** | Range |
| Age | 572 | (100%) | 40.5 (39.8-41.1) | 20-73 |
| Diagnostic age | 572 | (100%) | 32.8 (32.0-33.5) | 10-72 |
| Diagnostic years | 572 | (100%) | 7.8 (7.4-8.2) | 1-26 |
| Gender  Male  Female | 482  90 | (84.3%)  (15.7%) |  |  |
| **Stages of HIV infection**  Asymptomatic  Symptomatic  AIDS | 270  142  160 | (47.2%)  (24.8%)  (28%) |  |  |
| **Transmission categories**  IVDU  Homosexual/ Bisexual  Heterosexual  Other  Unknown | 113  266  114  5  74 | (19.7%)  (46.5%)  (20%)  (0.9%)  (12.9%) |  |  |
