# Supplementary material for: The economic costs and health-related quality of life of people with HIV/AIDS in the Canary Islands, Spain
Source: BMC Health Serv Res. 2009 Mar 30;9:55. doi: 10.1186/1472-6963-9-55 (PMC2670289; doi:10.1186/1472-6963-9-55)
Supplement: Additional file 2 — Table 2. Main characteristics of the sample for direct and indirect costs. [file 1472-6963-9-55-S2.doc]

## Table 2. Main characteristics of the sample for direct and indirect costs

|  | **Direct Costs Sample**  **n = 572** | **Indirect Costs sample**  **n = 241** |
| --- | --- | --- |
| Age | 40.56 (8.24) | 41.57 (8.58) |
| Female | 15.7% | 14.9% |
| Male | 84.3% | 85.1% |
| IVDU | 18.5% | 18.7% |
| Non IVDU | 81.5% | 81.3% |
| Asymptomatic | 47.2% | 47.3% |
| Symptomatic | 24.8% | 25.5% |
| AIDS | 28.0% | 27.2% |
| CD4<200 | 7.6% | 7.7% |
| 200<CD4<500 | 32.9% | 24.3% |
| CD4<500 | 59.4% | 68.0% |
| CVRS (EQ-5D Tariff) | 0.791 (0.273) | 0.777 (0.221) |

Percentages and mean (standard deviation between brackets)
