# Supplementary material for: The economic costs and health-related quality of life of people with HIV/AIDS in the Canary Islands, Spain
Source: BMC Health Serv Res. 2009 Mar 30;9:55. doi: 10.1186/1472-6963-9-55 (PMC2670289; doi:10.1186/1472-6963-9-55)
Supplement: Additional file 3 — Table 3. Mean Cost for people with HIV and AIDS in the Canary Islands, 2003. [file 1472-6963-9-55-S3.doc]

**Table 3. Mean Cost for people with HIV and AIDS in the Canary Islands, 2003**

|  | **Asymptomatic**  **n = 270** | | | | **Symptomatic**  **n = 142** | | | **AIDS**  **n = 160** | | |
| --- | --- | --- | --- | --- | --- | --- | --- | --- | --- | --- |
|  | **Values (€)** |  | |  | **Values (€)** |  |  | **Values (€)** |  |  |
|  |  | **% direct costs** | | **% total costs** |  | **% direct costs** | **% total costs** |  | **% direct costs** | **% total costs** |
| **Direct health-care costs (€)** |  |  | |  |  |  |  |  |  |  |
| Hospital care | 26 | 0.36% | | 0.25% | 262 | 3.08% | 1.81% | 1,084 | 11.01% | 6.88% |
| Medical visits | 240 | 3.36% | | 2.28% | 333 | 3.91% | 2.3% | 359 | 3.65% | 2.28% |
| Drugs (ART) | 6,120 | 85.62% | | 58.12% | 6,870 | 80.75% | 47.41% | 7,143 | 72.59% | 45.36% |
| Drugs (no ART) | 72 | 1.02% | | 0.69% | 199 | 2.34% | 1.38% | 307 | 3.12% | 1.95% |
| Medical tests | 645 | 9.02% | | 6.12% | 793 | 9.32% | 5.47% | 854 | 8.68% | 5.42% |
| Emergencies | 44 | 0.62% | | 0.42% | 51 | 0.6% | 0.35% | 90 | 0.91% | 0.57% |
| Rehabilitation | 0 | 0.00% | | 0.00% | 0 | 0.00% | 0.00% | 4 | 0.04% | 0.03% |
| **Total, direct costs (€)** | **7,148** | 100.00% | | 67.88% | **8,508** | 100.00% | 58.72% | **9,842** | 100.00% | 62.49% |
| **Indirect costs (morbidity) (€)** |  |  |  | |  |  |  |  |  |  |
| Indirect costs | 3,383 |  | 32.12% | | 5,981 |  | 41.28% | 5,908 |  | 37.51% |
| **Total costs (€)** | **10,531** |  | 100.00% | | **14,489** |  | 100.00% | **15,750** |  | 100.00% |

Units: euros, 2003

ART: Antirretroviral Therapy

NO ART: No Antirretroviral Therapy
