# Supplementary material for: The economic costs and health-related quality of life of people with HIV/AIDS in the Canary Islands, Spain
Source: BMC Health Serv Res. 2009 Mar 30;9:55. doi: 10.1186/1472-6963-9-55 (PMC2670289; doi:10.1186/1472-6963-9-55)
Supplement: Additional file 4 — Table 4. Direct Costs for people with HIV and AIDS in the Canary Islands (2003). [file 1472-6963-9-55-S4.doc]

**Table 4. Direct Costs for people with HIV/AIDS and CD4 in the Canary**

**Islands (2003)**

|  | **Type of expenditure** | | |  |
| --- | --- | --- | --- | --- |
| **Disease strata** | **Impatient** | **Outpatient** | **Medications** | **Total Direct Costs** |
| Asymptomatic HIV | 26 € | 930 € | 6,193 € | 7,148 € |
| Symptomatic HIV | 262 € | 1177 € | 7,069 € | 8,508 € |
| AIDS | 1,084 € | 1308 € | 7,451 € | 9,842 € |
| CD4 cell count >500 cell per μL | 132 € | 1,036 € | 6,770 € | 7,938 € |
| CD4 cell count 200-500 cell per μL | 786 € | 1,179 € | 6,734 € | 8,699 € |
| CD4 cell count <200 cell per μL | 647 € | 1,299 € | 7,355 € | 9,302 € |
