# Supplementary material for: The economic costs and health-related quality of life of people with HIV/AIDS in the Canary Islands, Spain
Source: BMC Health Serv Res. 2009 Mar 30;9:55. doi: 10.1186/1472-6963-9-55 (PMC2670289; doi:10.1186/1472-6963-9-55)
Supplement: Additional file 5 — Table 5. Mean scores for SF-36 and EQ-5D. [file 1472-6963-9-55-S5.doc]

**Table 5. Mean scores for SF-36 and EQ-5D**

| **SF-36 (scores)** | Disease Stage | **Mean (SD)** |
| --- | --- | --- |
| **PCS** | Asymptomatic HIV | 50.07 (10.55) |
|  | Symptomatic HIV | 47.44 (10.30) |
|  | AIDS | 46.07 (10.74) |
| **MCS** | Asymptomatic HIV | 39.52 (14.67) |
|  | Symptomatic HIV | 40.99 (13.24) |
|  | AIDS | 35.63 (13.88) |
| **EQ-5D (scores)** | Disease Stage | **Mean (SD)** |
| **EVA** | Asymptomatic HIV | 0.79 (0.21) |
|  | Symptomatic HIV | 0.80 (0.20) |
|  | AIDS | 0.73 (0.23) |
