# Supplementary material for: The economic costs and health-related quality of life of people with HIV/AIDS in the Canary Islands, Spain
Source: BMC Health Serv Res. 2009 Mar 30;9:55. doi: 10.1186/1472-6963-9-55 (PMC2670289; doi:10.1186/1472-6963-9-55)
Supplement: Additional file 6 — Table 6. Direct and indirect costs and quality of life. [file 1472-6963-9-55-S6.doc]

**Table 6. Direct and indirect costs and quality of life**

|  | **DIRECT COSTS** | **INDIRECT COSTS** |
| --- | --- | --- |
| **EQ-5D** | **Mean (SD)** | **Mean (SD)** |
| **Low QOL**  (score < 0,50**)** | 8,795 € (4,244) | 5,598 € (6,903) |
| **Medium QOL**  (score 0,50 – 0,75) | 8,861 € (4,835) | 6,176 € (8,457) |
| **High QOL**  (score > 0,75) | 7,770 € (3,917) | 4,247 € (6,671) |

QOL: quality of life

SD: standard deviation
